# Therapy of breast cancer patients with disorders of the anxiety-depressive spectrum

**DOI:** 10.1192/j.eurpsy.2021.1983

**Published:** 2021-08-13

**Authors:** T. Shushpanova, O. Shushpanova

**Affiliations:** 1 Clinical Psychoneuroimmunology And Neurobiology Lab, Mental Health Research Institute, Tomsk, Russian Federation; 2 Child Psychiatry, Mental Health Scientific Center, Moscow, Russian Federation

**Keywords:** oncology, breast cancer, anxiety-depressive spectrum

## Abstract

**Introduction:**

Breast cancer (BC) is one of the leading causes of cancer death worldwide. The problem of mental health and quality of life of patients is currently particularly relevant. Most patients with breast cancer in the process of adapting to the disease experience certain mental disorders: depressive, anxiety-phobic and psychosomatic disorders.

**Objectives:**

To study the severity of anxiety-depressive disorders in the clinical picture in patients with breast cancer and evaluate the effectiveness of specialized pharmacotherapy using antidepressants in combination with antitumor therapy.

**Methods:**

The study included 30 patients with a first established diagnosis of breast cancer and 52 patients with a follow-up history of 3-17 years. The main method of work was the clinical, psychopathological, and statistical research methods (a method using contingency tables and the Fechner coefficient, a method - Chi-square test).

**Results:**

To assess the severity in the clinical picture of anxiety-depressive tendencies and the effectiveness of treatment, special scales were used: hospital scale of anxiety and depression (HADS); general clinical impression scale (CGI) for assessing disease severity (CGI-S “severity”) and improvement (CGI-I “improvement”). High antidepressant therapy efficacy indicators were obtained in combination with benzodiazepine drugs and hypnotics in a group of patients with anxiety-depressive nosogenia (15 patients, 88% of respondents with reduction in starting anxiety and depression scores HADS more than 50%, CGI 85%), in the group with chronic hypochondriac dysthymia and cyclotymic endoform depression.

**Conclusions:**

The data obtained in the study confirm the effectiveness of psychopharmacotherapy with antidepressants in breast cancer patients with identified disorders of the anxiety-depressive spectrum.

**Disclosure:**

No significant relationships.

